# Supporting implementation science and health equity in cancer prevention and control through research networks

**DOI:** 10.1007/s10552-023-01732-9

**Published:** 2023-06-16

**Authors:** Prajakta Adsul, Stephanie B. Wheeler, Alexa L. Young, Rebecca J. Lee, Heather M. Brandt

**Affiliations:** 1 Department of Internal Medicine, School of Medicine, University of New Mexico, Albuquerque, NM USA; 2 Cancer Control and Population Sciences Research Program, University of New Mexico Comprehensive Cancer Center, Cancer Research Facility, Room G11, MSC07 4025, 1 University of New Mexico, Albuquerque, NM 87131-0001 USA; 3 Center for Health Promotion and Disease Prevention, University of North Carolina at Chapel Hill, Chapel Hill, NC USA; 4 Lineberger Comprehensive Cancer Center, University of North Carolina at Chapel Hill, Chapel Hill, NC USA; 5 Department of Health Policy and Management, Gillings School of Global Public Health, University of North Carolina at Chapel Hill, Chapel Hill, NC USA; 6 HPV Cancer Prevention Program and Department of Epidemiology and Cancer Control, St. Jude Children’s Research Hospital, Memphis, TN USA

**Keywords:** Cancer prevention, Cancer control, Health equity, Qualitative interviews, Networks, Cancer health disparities

## Abstract

The Cancer Prevention and Control Research Network (CPCRN) is a national network of academic, public health, and community organizational partners across multiple geographic sites who collaborate to reduce the cancer burden in diverse communities. Given key recommendations that suggest the need for cross-disciplinary collaboration in cancer prevention and control, we sought to explore the historical and contemporary evolution of health equity and disparities research as an area of focus within the CPCRN over time. We conducted 22 in-depth interviews with former and current leaders, co-investigators, and other members of the network. Several key themes emerged from data that were analyzed and interpreted using a constructivist, reflexive, thematic analysis approach. Nearly all participants reported a strong focus on studying health disparities since the inception of the CPCRN, which offered the network a distinct advantage in recent years for incorporating an intentional focus on health equity. Recent law enforcement injustices and the inequities observed during the COVID-19 pandemic have further invigorated network activities around health equity, such as development of a health equity-focused workgroup toolkit, among other cross-center activities. Several participants noted that, in terms of deep, meaningful, and impactful health equity-oriented research, there are still great strides for the network to make, while also acknowledging CPCRN as well-aligned with the national dialogue led by federal agency partners around health equity. Finally, several future directions were mentioned by the participants, including a focus on supporting a diverse workforce and engaging organizational partners and community members in equity-focused research. Findings from these interviews provide direction for the network in advancing the science in cancer prevention and control, with a strengthened focus on health equity.

**Supplementary Information:**

The online version contains supplementary material available at 10.1007/s10552-023-01732-9.

## Introduction

With accumulating evidence in recent years, cancer prevention and control research has increasingly incorporated a focus on dissemination and implementation of evidence-based interventions (e.g., cancer screenings, HPV vaccination, and lifestyle behavior change interventions) [1]. Such progress has been informed by approaches that include community-based participatory research [2], adaptation of interventions to different populations and settings [3], and work with clinical and community partners to improve implementation within healthcare and community settings [4]. Implementation science as a field benefits from these cross-disciplinary perspectives and multi-sector collaborations to ensure that research findings lead to population-level health outcomes [5].

Such collaborations can be difficult to configure within a single research institution or when led by a sole Principal Investigator. Research networks on the other hand, may bring together cross-disciplinary collaborations which are often well-equipped to overcome deficits or limitations that may otherwise exist within a single institution. Existing evidence reveals that researchers involved in networks generate high-quality work and that this work leads to overall higher quality of science through peer review and support within the network [6]. In addition, engaging community members in research ensures that the work is responsive to community needs and mindful of community constraints [7]. Participation in research networks also influences knowledge dissemination, collaboration, implementation, and policies [8], while providing increased access to innovative interventions for both investigators and community members alike [9].

One such research network is the Cancer Prevention and Control Research Network (CPCRN), a national network of academic, public health, and community organizational partners across multiple geographic sites that collaborate with the goal of reducing cancer burden in diverse communities. The CPCRN is a thematic research network of the Centers for Disease Control and Prevention (CDC) Prevention Research Centers (PRC) Program focused on preventing and controlling chronic diseases. Described as a “network of networks,” the CPCRN enables and conducts multicenter collaboration while leveraging expertise, resources, and partnerships [10]. Over the past 20 years, CPCRN members have collaborated across institutions and disciplines to conduct large-scale studies designed to accelerate the adoption, implementation and sustainment of evidence-based interventions for population health outcomes. A key strength of the network is in the study of cancer prevention and control implementation strategies through collaboration with community, clinical, and organizational partners. More specifically, CPCRN directs a concerted focus toward those who have been minoritized, marginalized, and medically underserved.

Cross-disciplinary collaborations in cancer prevention and control are needed for promoting a research agenda geared toward widespread population health and health equity [11]. To that end, we sought to explore the historical and contemporary evolution of CPCRN’s focus on health disparities and equity research and set future directions for the network, as well as the field at large, in advancing health equity in cancer prevention and control research.

## Methods

Specific details on recruitment and data collection methods are described elsewhere [12]. In brief, in-depth, semi-structured interviews were conducted with 22 leaders, investigators, and staff involved in the CPCRN network either in the past or currently. Interview questions focused on perspectives of those involved in the network about the structure, organization, processes, and outcomes employed by CPCRN to advance cancer prevention and control research, as well as specific questions about how and to what extent health disparities and equity had been important topics for network investigation over time. In the interviews, we used a semi-structured approach to explore the perceptions around health equity, providing us in-depth information. All interviews were conducted via Zoom and audio recorded. De-identified transcripts were used for analysis. For analyses, we were guided by Braun and Clarke’s thematic analysis methods and used a constructivist approach to examine the realities presented in the data [13]. Specific steps for analysis included: familiarizing with the interview transcripts, coding, generating themes, and re-checking themes back with the original data. In this brief report, we present the themes and supportive quotes to better understand the evolution of health equity research in cancer prevention and control research as supported by the leadership and investigators from the CPCRN.

## Results

**Fig. 1:**
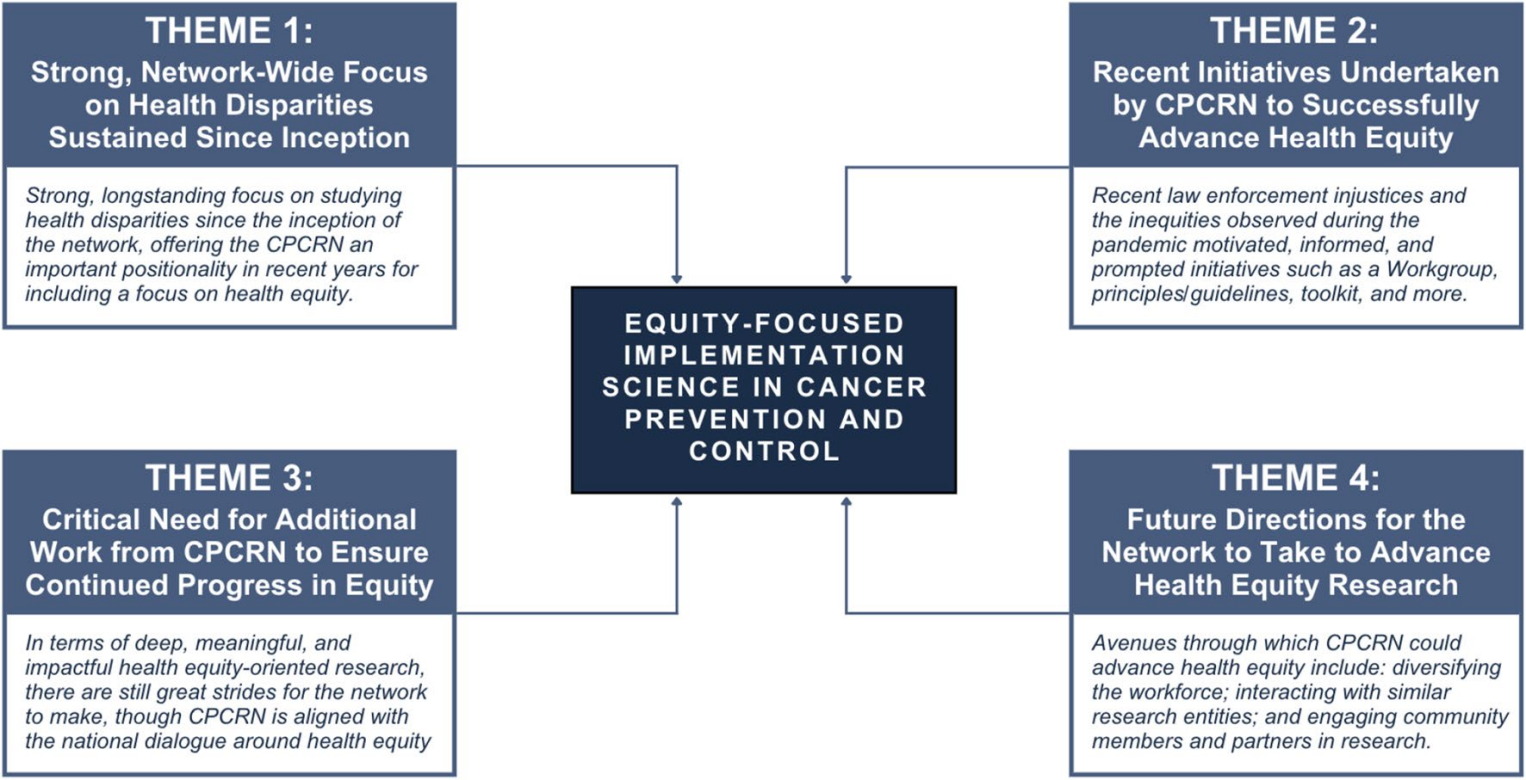
Thematic analysis breakdown

Participant characteristics are described elsewhere [12]. Briefly, using a convenience sample, we interviewed 22 current and former CPCRN representatives, comprised of collaborating center investigators, coordinating center members, federal agency partners, and academic and community affiliates. Among the participants, three were formerly engaged in the CPCRN, offering historical insight into the early years of the network. This, in conjunction with diverse perspectives collected across the 19 remaining interviewees currently active in the network, provided a comprehensive landscape of CPCRN, inclusive of voices from network inception to present. Below, we present our findings under four key themes with illustrative quotes that emerged from our analysis (also depicted and summarized in Fig. 1). Table 1 provides additional quotes from these interviews presented under each theme.

**Table 1:** Primary themes and corresponding, illustrative quotes about health equity as a focus of CPCRN

Themes	Illustrative quotes
Theme #1 Almost all participants reported a *strong, longstanding focus on studying health disparities* since the inception of the network, which offered CPCRN a distinct advantage in recent years for incorporating an intentional focus on health equity	“…*I know that when we called it different things were public health disparities reducing health disparities… the idea that a focus needs to on improving…increasing the research related to cancer control implementation among minority and other underserved populations, I think that’s always been there.” (Participant 21)* *“I think … we pay attention to it, and we’re always thinking about which people and groups have been minoritized, marginalized, excluded, and discriminated against. You know, thinking “Who are those people and what are those groups who are much less likely to have access to cancer prevention and control? How can we understand how to reach them effectively?” I think that’s always been a guiding mantra in this Network.” (Participant 15)* *“I don’t remember [health equity] ever being an explicit focus. I think, our conversations about health equity have really only become explicit, and now, to the fore, in the field, since the murder of George Floyd, even though there were individuals working in health equity, uh, within implementation science prior to that. Um, but implicitly, it certainly was there. I mean, many of the projects were specifically focused on supporting the D&I of evidence-based interventions (EBIs) to vulnerable populations… So, I think there was a strong interest in disparities and reducing disparities by increasing access to EBIs. But I don’t know that health equity was an explicit discussion topic [within the Network]. If we talked about it, we talked about in terms of disparities and disparities-reduction.” (Participant 10)*
Theme #2 Recent law enforcement injustices and the inequities observed during the pandemic *further motivated, informed, and prompted new health disparities and equity initiatives*, such as development of a Workgroup, principles, toolkit, and more in the network	*“One key thing that you always hope a Network like this can do is be really responsive to the times… I don’t know that these conversations are happening, but I would be very surprised if they’re not–we now have this enormous problem with missed cancer screenings because of the pandemic…and this Network seems well-poised to address that.” (Participant 16)* *“It's definitely more explicit now than it has been [in the past], in terms–it's being more explicitly and consistently addressed. I think that, previously, it's been an explicit charge in terms of it being, I think, explicit in the mission of the Network and the vision of the network. But I think that it's different now in terms of the normalization of it in terms of the creation of the principles and guidance to help ensure that consistently across our efforts, this is being acknowledged, so the way that we address health equity is being done in a consistent way. That is different from previous cycles, and I think what prompted that certainly is, you know, world events have helped to accelerate it; how explicit we are in those efforts.” (Participant 20)* *“CPCRN is thinking at multiple levels, thinking about how systems and structures impact equitable access to cancer prevention and control. And now, we’ve evolved in our field to have more understanding about what that looks like, operationally from an implementation perspective, because it's no longer sufficient to say, “Our work is addressing equity issues” just because you’re working with a population that has historically and currently experienced those inequities.” (Participant 15)*
Theme #3 Several participants noted that in terms of *deep, meaningful, and impactful health equity-oriented research*, there are still great strides for the network to make, while noting that the network was aligned with the national dialogue around health equity	*“…there are other networks that are a few steps removed from thinking, "How does this drive public health? How does this improve cancer prevention and improve cancer control?" And I think CPCRN’s focus on what matters is really important. And what that means is that CPCRN is then better positioned to be thinking about some of the key determinants of how folks fare throughout this cancer journey, right?” (Participant 13)* *"I just think CPCRN is a great forum to be able to push some of those discussions through, you know, get speakers, training, things that would really help push the implementation science field a little bit further in that direction [toward health equity].” (Participant 11)* *“But I think it’s important for us to know, to understand better maybe for me and others to learn better, if we want to center health equity is part of our work. Are we really doing that as a network? Are we doing it on one off projects?” (Participant 1)*
Theme #4 Several future directions were mentioned by the participants, including a focus on *supporting a diverse workforce, interactions with similar research networks, and engaging community members and partners* in research	*“I think we’ve done quite a bit in terms of, you know, thinking about how we engage with clinics or communities. How do we do our research? What are our methods? I think we could do more and be more intentional about innovation around methods.” (Participant 11)* *“…just because you have [different] skin colors represented doesn’t mean you have all the tribal perspectives represented; you don’t have all the different diversity of thinking. So, I think that we become more diverse…it’s more the diversity of perspectives, people with different orientations and ways of thinking about things. And that's, in my mind, a good thing…” (Participant 9)* *“The fact of the matter is that people who are the senior level [investigators] who’ve been around for a long time, they’re white and they're mostly women. So, you move that needle by, when they move on and they retire, you want to bring the younger generation in and mentor them so that when you leave, you are leaving a more diverse, kind of, supported cohort of researchers who better represent the communities that we're working in.” (Participant 8)* *“A network like CPCRN can come together and make a difference in [the health equity] space, not only with its words, but in its actions as well. I think the whole notion of the [CPCRN] Scholars Program and wanting to support junior faculty of all backgrounds…a diverse group, that’s what we’re looking for; a diverse group of faculty scholars to take advantage of the mentorship and training opportunities that we have in the Network.” (Participant 2)*

### Theme 1: Longstanding focus on health disparities

Almost all participants reported a strong, longstanding focus on the study of health disparities; a priority area of CPCRN dating back to network inception and offering CPCRN a distinct advantage in recent years to place an intentional focus on health equity.

Several participants that were engaged in the initial years of the CPCRN mentioned the unique federal funding structure of the network (i.e., from a partnership between the CDC Division of Cancer Prevention and Control and the National Cancer Institute’s (NCI) Community Networks Program though the Center to Reduce Cancer Health Disparities), which helped to center the early CPCRN research focus on health disparities reduction. As one participant noted, an early focus on populations experiencing disparities reflected urgent and pressing public health and scientific needs for cancer prevention and control research:

*I felt pretty strongly that that was where we could contribute [to CPCRN], and from a scientific perspective at that, because that's where the action is. I mean, you go to where disparities are, because that's where the scientific action is. But, it's also where the public health action is, that's where we're needed the most. (Participant 9)*

Participants also mentioned that understanding and reducing cancer health disparities has always been a central theme for the network, as demonstrated in the research prioritized through Workgroups focused on rural health, Federally Qualified Health Centers (often referred to as FQHC’s), and access to care among minoritized and underserved populations. Especially in the last decade, participants noted a heightened research focus by CPCRN on social determinants of health, social needs, and the use of community-based participatory research (CBPR) approaches in cancer prevention and control research. A key strength of the network was the explicit focus on community-oriented research that emphasizes translation of research findings as they are relevant to the community’s priorities, as mentioned by one participant:

*…behind all of the CPCRN work is this focus on the community, and it's become really apparent in this funding cycle; disparities and a focus on the community. So, what I love is that [the Network doesn't] expect us to constantly do, you know, randomized clinical trials, but rather, they actually want us to do development, they want us to look at community priorities. They don't scoff at qualitative mixed-methods work, they see and understand the value of implementation science and implementation practice, which is how you speed up the translation of research to practice. (Participant 10)*

Participants also noted that the network has supported investigators in advancing the science around cancer health disparities through high quality research. Specifically, one individual expressed the sentiment that a consistent focus on health disparities over time has paved the way for an incorporate a focus on health equity in more recent years:

*I think we were very quickly able to pivot, because [a health disparity focus] was already there. We didn’t have to change our research agenda all that much. We just had to pay much more attention, word things a little bit differently, maybe prioritize things a little bit more. I think the pivot [to health equity] is feeling very smooth to me, unlike some other projects where health disparities [focus] is not even part of the picture. (Participant 8)*

### Theme 2: New initiatives and resources are critical for continued for advancements in health equity

Recent law enforcement injustices and the inequities observed during the pandemic further motivated, informed, and prompted new health disparities and equity initiatives, such as development of a Workgroup, principles, toolkit, and more in the network.

As described before, CPCRN investigators noted a strong focus on health disparities throughout the multiple years of research supported by the network. However, the recent law enforcement injustices (i.e., the murder of Mr. George Floyd and injustices toward members of the Black communities across the US) and the inequities observed in the health outcomes for minoritized communities during the COVID-19 pandemic, inspired CPCRN members to focus more explicitly on research around health equity. Some participants clarified this focus on health equity as follows:

*...at the very beginning, the entire focus of CPCRN was on addressing health disparities. So, I think I’'s just a natural evolution that the Network has moved towards health equity, though, I think, all along, [health equity] is something that, at the base, was built into CPCRN. Health equity is getting a lot more attention now [beyond the network] in a way that is distinct [from health disparities]; you know, there’s nuances between [the two] … (Participant 3)*

*I do think that terminology is important. And I think that, while those of us who’ve been working in this space, even way back when it was called “minority health” have always understood that there is an equity component, like it’s a social, political, sort of component to this problem of inequities. But I think the word makes it more obvious that we’re talking about, not just differences, but differences that are caused by underlying discrimination, social systems, racism, and so, I think it’s important. (Participant 21)*

Many participants cited the creation of a Workgroup specifically focused on health equity, as well as the amendment of the CPCRN mission and vision statements to reflect a prominent health equity focus, as particularly important initiatives undertaken by the network in recent years.

### Theme 3: Critical growth and action remain for CPCRN to advance health equity

Several participants noted that in terms of deep, meaningful, and impactful health equity-oriented research, there are still great strides for the network to make, while noting that the network was aligned with the national dialogue around health equity.

Study participants identified several characteristics of the network and its investigators, that they perceived to be particularly noteworthy strengths that contributed to advancing the science of cancer prevention and control, including research that focuses on multiple socioecological levels beyond the individual, use of community-based participatory approaches, and alignment of research interests and expertise with community priorities. A few participants cautioned that operationalizing a focus on health equity and building it into research designs will warrant newer approaches and thinking, while being mindful not to exacerbate disparities. In terms of implementation science studies, some partners noted the need for a specific focus on inequities, as noted below:

*So, we’ve talked a lot about implementation… so people tend to have this idea that you [should] go work with the people and places who are already doing pretty well, and you work out how to help them to do better. While it may be a good thing for them, if equity is the goal, just focusing on those who are already sufficiently ready (pauses), um, means that we may not make as much progress as we would like, and it means that we’re already ruling out a lot of really important settings or questions that we need to ask. So, CPCRN, I think, would do well, I think we would all do well over time, to just be very explicit about, 'Who are we engaging? Who are we not engaging? And what does that say about our prospects in making a benefit across the country?’ (Participant 13)*

Claiming a health disparities-focused research agenda was considered insufficient; participants felt strongly that real change comes from a commitment to health equity by holding each other accountable to meaningfully incorporate health equity into the CPCRN research agenda. Participants noted that addressing social determinants of health would not be easy in any five-year grant cycle or through research alone. However, the multi-site collaborative nature of CPCRN and investigators with diverse experiences provides the opportunity for the network to measure, understand, and design interventions that can take on key structural barriers to health across diverse settings. One participant detailed a Venn Diagram to illustrate the importance of research networks in promoting capacity building and sustainability, as follows:

*I think it's that Venn diagram…where you have the CPCRN, the Network, and then you have your [community] partners and then the researchers, and there's that sweet spot in the middle where you have the chance to really focus on building capacity and individual agency designing for sustainability, integration of equity considerations from the beginning and acting in a way that's going to recognize and help ensure that what's getting done isn’t going to continue to exclude some...and thinking about the resources that are available. Like, in my mind, that's really the beauty*. *(Participant 15)*

Through these approaches, many participants perceived the network to be responsive to the contemporary challenges in incorporating health equity, and encouraged the intentional efforts toward health equity, moving beyond geographical access to examining the structural determinants of health that impact an individual’s cancer journey.

### THEME 4*:* Future directions

Finally, several future directions were mentioned by the participants, including a focus on supporting a diverse workforce, interactions with similar research networks, and engaging community members and partners in research.

Several participants mentioned the network’s responsiveness to contemporary world events, among other strategies undertaken to incorporate a focus on health equity, including submissions to recent Requests for Information from federal agencies, developing resources with a principle focus on health equity, and engaging in ongoing introspection through the current funding cycle. In focusing on health equity, several participants referenced a lack of diversity in the network, which they went on to note has not been historically tracked by CPCRN. At recent network meetings, participants also observed very few people of color in a room full of white women, and mentioned the need to be reflective of positionality and to acknowledge the current state, as described by the following participant:

*I really was struck at our most recent [CPCRN Annual] Meeting where there was collective observation about, like, ‘Look around the room and what does the room look like?’ And, you know, the room was mostly white, the room was not as diverse as we would like it to be. And so, there's many reasons for that right now, but how can we be more intentional in engaging other voices that aren’t perhaps represented? And I think we have a ton of opportunities to grow in that area and see [health equity] even further integrated into the activities. (Participant 15)*

Many noted the crucial role of supporting the next generation of diverse scholars through the ongoing activities of the CPCRN Scholars Workgroup, which is currently in its third year and has drawn a highly diverse group of early career investigators to the network.

While committing to health equity within the network, participants also mentioned the value of cross-network collaboration with entities similarly focused on health equity, including the working group focused on health equity at the Consortium of Cancer Implementation Science, as one Federal Agency partner noted:

…*CPCRN is interconnected with other groups that are trying to advance similar missions…there’s an intended big tent approach, which is inclusive and certainly has involved leadership roles from folks at CPCRN. But yeah, looking at who we are as a Network and how we can practice what we preach is incredibly important if our goal is to be more engaging of a broader, more diverse community beyond the network. (Participant 13)*

Investigators also noted the important role of supporting community outreach and engagement offices at NCI-designated cancer centers:

*…For community outreach and engagement (COE) [efforts] within cancer centers, I think that CPCRN, definitely, whether we’re the leader of that, or we’re helping, you know, with our cancer centers in that area–I know, a lot of different investigators might be the main PI or the lead or director of that at their cancer centers, … but we’re heavily involved, you know, on their advisory board and involved in other implementation science support. (Participant 4)*

Others also mentioned engaging members from historically diverse institutions and other minority-serving organizations, as noted below:

*I think there’s always been this focus on medically-underserved populations, I think now, [the network is] really starting to move in that direction, and even more than they were before… But like really trying to focus the efforts more in that direction I think [would be] great. One suggestion during the [CPCRN Annual] meeting was including more diverse investigators in the network, because the institutions that are awarded, you know, our larger [collaborating centers] are all research institutions, so there are a lot of other institutions [to consider engaging] like Historically Black Colleges and Universities (HBCUs), Hispanic-serving organizations, and I think someone maybe even mentioned American Indian[-serving]. (Participant 7)*

## Discussion

Continuing the growing momentum to addressing health inequities and promoting health equity will require us to join efforts beyond the capacities for individual researchers and research institutions. In this qualitative analysis of 22 interviews of people involved in CPCRN over a 20-year period, a clear focus on health disparities and health equity research was noted, with important and timely opportunities to grow and diversify further both internally as a network and within the research portfolio. Almost all participants reported on CPCRN’s strong focus on studying health disparities since the inception of the CPCRN, which, in more recent years, expanded to include health equity as well. Recent critical action steps were noted in establishing a formal Health Equity Workgroup and the development of health equity principles and a toolkit for researchers [14–16]. To promote deep, meaningful, and impactful health equity-oriented research, participants noted key future strategic directions which included supporting a diverse workforce and building stronger collaborations across research networks.

One of the challenges, noted widely in the contemporary literature, is the transition between health disparities focused research to the focus on equity [17]. In implementation science there are continuing calls for grounding the science in health equity, using an anti-racist lens, and addressing structural racism [11, 18, 19]. As noted with urgency, scholars have called for a shift toward health equity through development and implementation of interventions at the neighborhood, local, community, state, and national levels, when considering population level benefit [20]. Working on multiple socioecological levels adds complexity, which requires cross-disciplinary collaboration within investigators and also with partners serving populations and members of the populations experiencing health inequities. Participants in this study demonstrated this refined understanding, that recognized these differences and suggested concrete next steps to continue the focus of the network on health equity.

What was also clear from the data is a strong commitment, among CPCRN participants, to serving groups who have been or are currently marginalized. Marginalized and medically underserved populations are often least likely to be up-to-date with prevention and control recommendations and also suffer from higher cancer incidence and mortality [1]. The reasons for these disparities are complex, multilevel, and deeply rooted in historical and contemporary racism, classism, sexism, ageism, and ableism. This research network offers a structure, a means of interacting and collaborating, and proven pathways that illustrate how geographically dispersed people can work together to solve complex problems in partnership with others who care deeply about the failures in structure and processes that lead to disparate outcomes. The CPCRN is comprised of investigators that share a common purpose, which in turn, amplifies the magnitude of influence and potential of combined efforts to benefit diverse populations. Other research networks may adopt this model of high levels of multi-disciplinary, cross-institution collaboration centered around addressing cancer-related health disparities and broader health equity issues. Research networks should strive for practices the fully integrate and optimize pursuits of health equity, rather than health equity tourism [21].

Study characteristics that we acknowledge to be limitations of note include modest sample size, utilization of a convenience sampling approach, and consideration for the possibility that participants who opted to take part may be those who are deeply committed to addressing cancer disparities. Nonetheless, this study brings considerable strengths, namely through the inclusion of diverse perspectives, with all representing all active years covering the full life of the network, as well as across different types of involvement, allowing the team to comprehensively explore the concept of how network attend to health equity.

## Conclusion

This study demonstrates the power of collaboration through long-standing and consistently funded research networks like the CPCRN across geographic locations and using a team science approach for addressing cancer disparities in prevention and control. We highlight challenges and opportunities for research networks in reducing cancer disparities and promoting health equity and reducing health equity tourism. CPCRN offers space for attending to the urgency of these issues in cancer prevention and control. We must use what we know works and determine the best ways to ensure that everyone benefits with special attention to people who have been excluded from mainstream population-level cancer prevention and control efforts—or have not been a priority. If we do not act with urgency, we jeopardize the potential benefits of applying evidence-based interventions and will exacerbate disparities.

### Supplementary Information

Below is the link to the electronic supplementary material.

Supplementary file1 (DOCX 13 kb)

## Data Availability

The data generated and analyzed during the current study will be made available through UNC Chapel Hill’s CPCRN Dataverse account.
