# Supplementary material for: Supporting implementation science and health equity in cancer prevention and control through research networks
Source: Cancer Causes Control. 2023 Jun 16;34(Suppl 1):35–44. doi: 10.1007/s10552-023-01732-9 (PMC10689544; doi:10.1007/s10552-023-01732-9)
Supplement: Supplementary file 1 — Supplementary file1 (DOCX 13 kb) [file 10552_2023_1732_MOESM1_ESM.docx]

**Supplemental Table 1** *Codebook*

| **CODE** | **OPERATIONALIZATION** |
| --- | --- |
| Commitment to  health disparities | Participant’s descriptions of how CPCRN has been committed to the health disparities agenda in cancer prevention and control. Also includes discussions around the rationale for the founding of such a network. |
| Intentional focus  on health equity | Descriptions from participants that tie the ongoing focus on health equity to network activities and engagement across investigators and partners. Also includes descriptions from participants which highlight the focus on health equity, as it evolved from a singular focus on health disparities. |
| Meaningful pursuit  of health equity | Participant’s perceptions regarding how a focus of health equity has or has not been incorporated into network-wide activities and research. |
| Role of other  research networks | Description of participant’s perceptions about the role of research networks such as CPCRN, since its inception and in the future. Also includes relationship of CPCRN with other federally-funded research networks and discussions around alignment of network priorities with the national focus on health equity. |
| Role of community members/partners  in CPCRN research | Discussions around the ongoing research in CPCRN that supports the involvement of communities through community based participatory research approaches, and clinical partners in the research. |
| Diversifying the workforce | Perceptions of the interviewees around the current diversity of the network and ideas to strengthen the diversity in the future. |
| Future directions  for the network | Overlaps with previous two codes (i.e., role of community members and partners, and diversifying the workforce) and incorporates any additional discussions towards future directions of the network. |
